# Optimizing nitrogen topdressing for winter wheat by coupling remote sensing data with the DSSAT model

**DOI:** 10.3389/fpls.2025.1658254

**Published:** 2025-11-21

**Authors:** Yu Zhao, Zeyang Wen, Chao Wang, Lujie Xiao, Zhenhai Li, Haikuan Feng, Guoqiang Li, Wude Yang, Meichen Feng

**Affiliations:** 1 College of Agriculture, Shanxi Agricultural University, Key Laboratory of Sustainable Dryland Agriculture of Shanxi Province, Taiyuan, China; 2 Institute of Agricultural Economics and Information, Henan Academy of Agricultural Sciences, Zhengzhou, Henan, China; 3 Key Laboratory of Quantitative Remote Sensing in Agriculture of Ministry of Agriculture and Rural Affairs, Information Technology Research Center, Beijing Academy of Agriculture and Forestry Sciences, Beijing, China

**Keywords:** winter wheat, data assimilation, nitrogen topdressing, remote sensing, crop growth model

## Abstract

**Introduction:**

Excessive fertilization not only causes environmental pollution and degrades water and soil quality but also increases production costs and reduces agricultural sustainability.

**Methods:**

Based on two consecutive years of field experiments, this study developed a two-step data assimilation strategy for nitrogen (N) topdressing recommendations for winter wheat. First, a data assimilation system was established by minimising the discrepancy between aboveground dry biomass (AGB) estimated from remote sensing and that simulated by the crop growth model using a particle swarm optimization approach. Second, target yields under varying growth conditions were constructed using the DSSAT model and N economic return curves to enable optimised N fertilization recommendations.

**Results:**

AGB monitoring model was developed, achieving satisfactory results in both the calibration and validation datasets, with determination coefficient (R²) (normalised root mean square error (nRMSE)) values of 0.94 (13.62%) and 0.82 (15.42%), respectively. Based on the data assimilation system, the data assimilation stability for AGB and yield are relatively high. The nRMSE values for AGB are 11.20% and 19.44% for the training and validation datasets, respectively. The nRMSE values for yield are 6.35% and 11.22% for the training and validation datasets, respectively. The data assimilation-based recommended fertilization shows a negative power-law relationship with AGB at the jointing stage (R² = 0.65). Under different yield levels, fertilization was reduced by 6.69%–34.08% compared with that under high yield levels.

**Conclusion:**

This study balances yield and production costs by developing a data assimilation strategy for N fertilization recommendations, which can maintain high productivity and sustainability.

## 1 Introduction

Winter wheat is one of the most important staple crops worldwide, and its yield is highly dependent on nitrogen (N) availability. Urea, the most widely used N fertilizer globally (Eickhout et al., 2006), tends to cause a temporal mismatch between N supply and crop demand due to its rapid dissolution (Liang et al., 2017; Li et al., 2015; Shakoor et al., 2018). Split applications of urea can improve the synchronization between N availability and crop demand, but the number of applications is often limited by practical and labor constraints (Zhao et al., 2013). Winter wheat has two critical periods of N demand, occurring at the seedling and jointing stages, with substantially higher requirements during the latter (Ma et al., 2021). Therefore, optimizing N topdressing during the middle and late growth stages is critical for maximizing crop growth and minimizing environmental impacts, thereby supporting sustainable agricultural development (Cui et al., 2018; Gu et al., 2013; Zhang et al., 2020).

The fundamental principle of precision N fertilization is to quantify the N status gap between the target field and an N reference plot, and to derive the appropriate N application rate based on nutrient balance principles (Morris et al., 2018). By acquiring crop canopy information and soil properties through remote sensing, key intermediate variables can be obtained to inform nitrogen fertilization recommendations and enhance the precision of fertilization decisions (Yu et al., 2024; Yue et al., 2024). N recommendation methods include direct and indirect approaches: direct methods estimate the optimal N rate directly through models or algorithms (Qin et al., 2018, Ransom et al., 2019), while indirect methods first predict intermediate variables such as yield or agronomic parameters and then infer the appropriate N rate (Raun et al., 2002; Colaco and Bramley, 2018; Wang et al., 2025). Remote sensing technology possesses advantages of rapid, non-destructive data acquisition and broad spatial coverage, making it an effective tool for achieving more scientific N fertilization and crop nutrient management (Zhao et al., 2021; Si et al., 2021; Li et al., 2024a). Remote sensing has advantages in capturing intermediate variables such as yield or agronomic parameters, N fertilization recommendations based on remote sensing data often adopt indirect methods. N fertilization models relying exclusively on vegetation indices are typically built on empirical formulas and often fail to fully capture the dynamic influences of environmental factors, soil N supply capacity, and climate variability on crop N demand (Raun et al., 2002; Zhao et al., 2019; Zhang et al., 2023). Crop growth models integrate environmental factors such as climate, soil, and water to dynamically simulate changes in crop N demand, exhibiting strong mechanistic basis and environmental adaptability. The incorporation of remote sensing information into crop growth models through data assimilation systems have emerged as a crucial technological approach to enhance the precision and intelligence of N fertilizer application strategy (Jin et al., 2024; Wang et al., 2024). The fundamental advantage lies in bridging the gap between observational data and process mechanisms, improving the precision of N demand estimation and the dynamic simulation and prediction of soil nutrient dynamics (Luo et al., 2023; Huang et al., 2024). Data assimilation combines the advantages of remote sensing data and crop growth models to enable precise decision-making and dynamic management for N fertilization recommendations (Silva et al., 2023). Li et al. (2024a) optimized N fertilization during rice tillering by integrating UAV remote sensing with the WOFOST model, reducing fertilizer use by 5.9%. Wang et al. (2024) optimized N fertilization using a data assimilation algorithm, reducing N use by 37.9%–61.2%, increasing profit, and providing a suitable approach for smart fertilization management. Although data assimilation techniques have been applied in precision fertilization research, most existing studies rely predominantly on average yield as the basis for application (Li et al., 2024b; Morari et al., 2021). N fertilization recommendation algorithm that use average yield fail to capture field and crop growth variability and overlook cost-benefit trade-offs, ultimately constraining N management’s economic performance. Therefore, it is necessary to develop a target yield model that considers field variability and economic efficiency to optimize data assimilation fertilization algorithms.

Since crop yield largely depends on aboveground dry biomass (AGB) accumulation, the accuracy of AGB monitoring using remote sensing directly influences the precision of yield prediction. Li et al. (2024) demonstrated that data assimilation systems using AGB as the state variable have advantages in fertilization recommendations. This study aims (1) to develop a wheat aboveground AGB (AGB) inversion model using hyperspectral data from the wheat canopy and AGB data, enabling rapid acquisition of wheat AGB information, (2) to integrate crop growth models and remote sensing data to construct a crop-specific fertilization recommendation algorithm, and (3) to evaluate the performance of data assimilation in N fertilization recommendation, assessing their potential for precision fertilization and improving wheat yield.

## 2 Materials and methods

### 2.1 Experimental design

**Figure 1:**
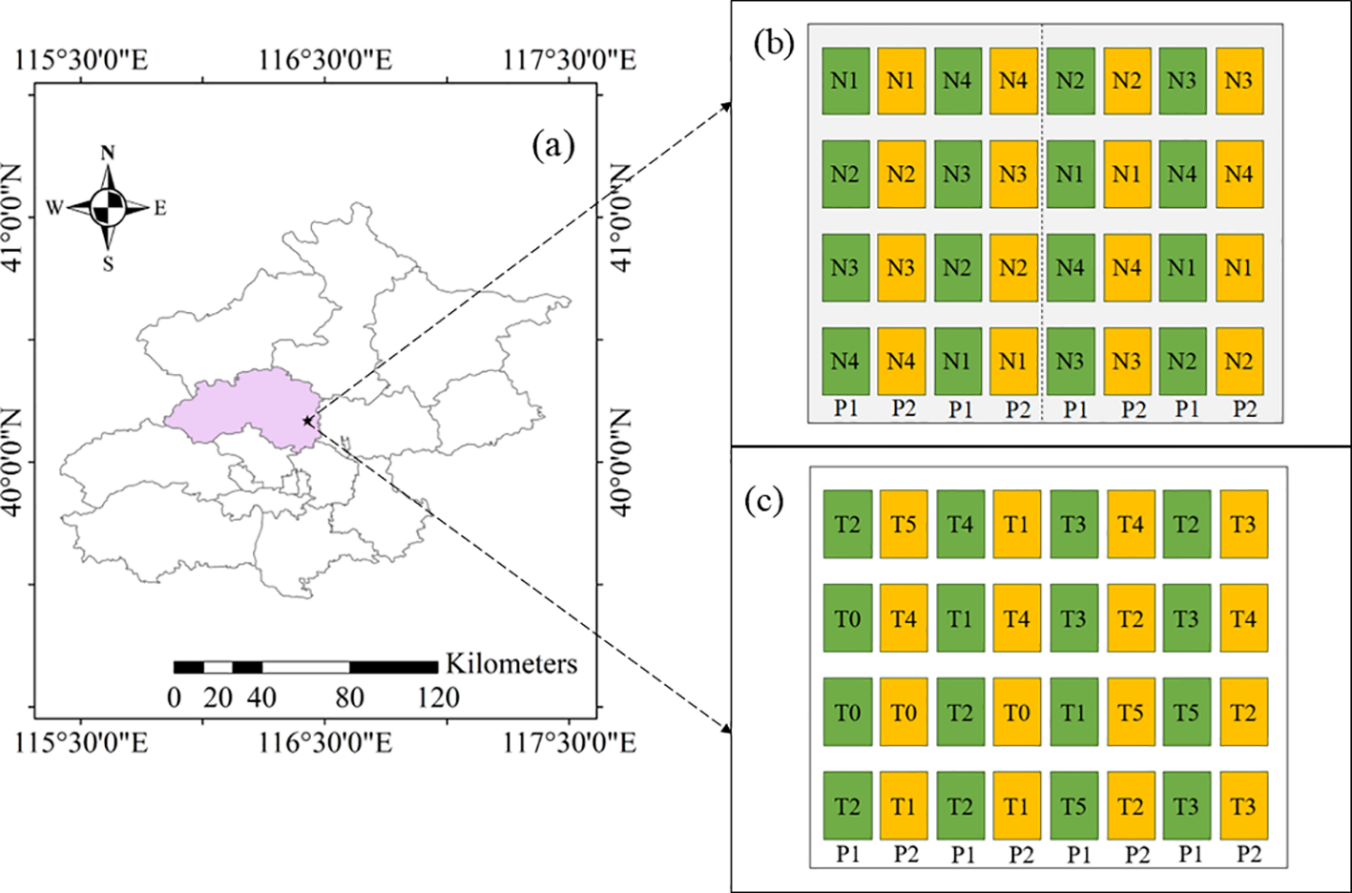
Locations of winter wheat experimental area **(a)** and experimental design [**(b)** Plot I: Modeling experimental plot; **(c)** Plot II: Topdressing experimental plot. P, N and T represent different varieties, N rates and N topdressing treatment, respectively.

This study was implemented during 2015–2018 at the Xiaotangshan National Precision Agriculture Research Centre (40.17°N, 116.43°E), positioned on the outskirts of Beijing, China (**Figure 1a**), a site well-known for precision farming research. The local site, characterized with warm temperate and semi-humid climate with a continental monsoon pattern, features silt loam soil with 0–30-cm-layer properties with concentrations of NO_3_^−^-N (3.16–14.82 mg kg^−^¹), NH_4_^+^-N (8.12–14.52 mg kg^−^¹), organic matter (15.8–20.0 g kg^−^¹), available phosphorus (3.14–21.18 mg kg^−^¹) and exchangeable potassium (86.83–120.62 mg kg^−^¹). The maximum temperature is 38.8 °C in summer, and the minimum temperature is -20.5 °C in winter. The annual amount of solar radiation and precipitation is 4850 MJ m^−2^, and 400–620 mm respectively (China Meteorological Data Service, http://data.cma.cn/).

Experiments 1 (2015–2016) and 2 (2017–2018) (**Figure 1b**) were identically designed as two-factor randomized complete block designs. The two winter wheat varieties tested were Luanxuan 167 and Jingdong 18. N application rates included four levels: 0 kg N ha^−^¹ (N1), 90 kg N ha^−^¹ (N2), 180 kg N ha^−^¹ (N3) and 270 kg N ha^−^¹ (N4). Urea was used as the N source and applied in equal amounts during the seeding and jointing stages. All other management practices adhered to local agricultural standards. There were 32 plots per growing season, with 128 biomass samples (32 plots × 4 periods) and 32 yields collected annually. The data from Experiments 1 and 2 were used for parameter calibration of the recommended fertilization model in this study. These data was used to set up DSSAT simulations with different fertilization rates during the jointing stage for target yield analysis.

Experiment 3 (2017–2018) was conducted during the 2017–2018 growing season, testing two wheat varieties (Luanxuan 167 and Jingdong 18). A base application of 90 kg N ha^−^¹ of N fertilizer was applied, with subsequent topdressing based on a data assimilation system during the jointing stage: T0 (no topdressing), T1 (25% of the recommended rate), T2 (50%), T3 (75%), T4 (100%) and T5 (125%). There were 32 plots with 128 biomass samples (32 plots × 4 periods) and 32 yields collected. Base fertilizers included 375 kg ha^−^¹ of calcium superphosphate and 150 kg ha^−^¹ of potassium sulfate. All other agronomic practices followed local farming standards. Experiment 3 was used for model validation.

### 2.2 Obtaining agricultural parameters of winter wheat

AGB measurements were conducted at key growth stages of winter wheat: stem elongation, flag leaf emergence, flowering, and grain filling. At each developmental stage, 20 stems of winter wheat were randomly selected from every experimental plot. After isolating the individual plant parts, the stems underwent a 30-minute heat treatment at 105 °C, followed by oven drying at 80 °C until reaching a stable mass. The final dry mass of each sample was then recorded. The aboveground dry biomass (AGB, t ha^-1^) was determined based on the stem count per hectare at each growth phase using the Equation 1:

(1) AGB=(D×n×15)/20

where AGB refers to aboveground dry biomass measured in tons per hectare, D indicates the dry mass in grams, and *n* stands for the number of stems per hectare across different growth stages. The values 15 and 20 represent the conversion factors for per-mu and per-hectare, and the conversion factor for dry weight to above-ground dry biomass, respectively (Zhao et al., 2025).

At harvest, a 1 m² standard sampling plot was established within the experimental field. All plants within the plot were harvested, and the grain dry weight was determined after threshing and oven drying. Afterward, the yield was normalized to a moisture content of 14% and converted to a per-hectare value (t·ha^−1^).

### 2.3 Meteorological data collection

Meteorological data, including daily precipitation and maximum and minimum temperatures, were obtained from the China Meteorological Data Sharing Service System (CMDSSS, https://data.cma.cn). Solar radiation was calculated using the Angström–Prescott formula described by Allen et al. (1998), based on the sunshine duration recorded in CMDSSS.

### 2.4 Remote sensing data acquisition

**Figure 2:**
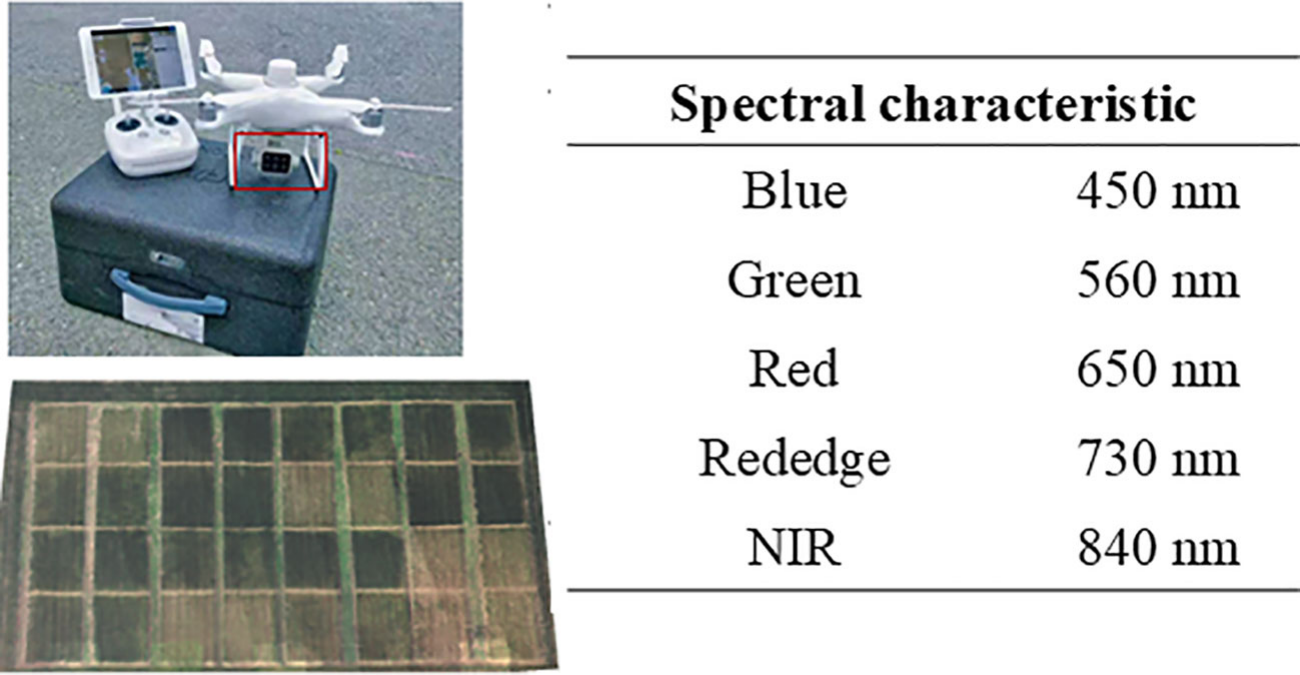
UAV platform equipped with multispectral camera.

A quadcopter UAV (Phantom 4 Pro, DJI, China) was used to collect remote sensing data under favorable weather conditions, specifically clear skies, no wind, and no cloud cover, with all flights adhering to the same take-off points and flight paths to ensure uniformity. Missions were conducted at 12:00 PM, maintaining a flight altitude of 30 m, with 80% forward and 85% lateral overlap. Prior to image acquisition, reflectance data from a spectral calibration panel were recorded to correct the pixel brightness values in the multispectral images. Orthomosaics were generated using Pix4Dmapper 4.3. Additional details on the image processing workflow can be found in Yang et al. (2021). The spectral characteristics of the sensor are illustrated in **Figure 2b**.

### 2.5 Selection of vegetation indices

In this study, the enhanced vegetation index 2 (EVI2), a widely adopted spectral index in vegetation monitoring, was employed to estimate AGB, following the formulation proposed by Jiang et al. (2008). The index is calculated using Equation 2:

(2) $$EVI2=2.5\;\times \;\frac{NIR-R}{NIR\;+\;2.4\;\times \;R\;+\;1}$$

where NIR and R are the reflectance values in the near-infrared (NIR) band (840 nm) and red band (650 nm), respectively. The constants 2.5, 2.4 and 1 are the correction factors to mitigate the influence of soil background, atmospheric interference and signal saturation, thereby enhancing the sensitivity of the index in densely vegetated areas.

### 2.6 Data assimilation framework for optimizing N application using the DSSAT model

**Figure 3:**
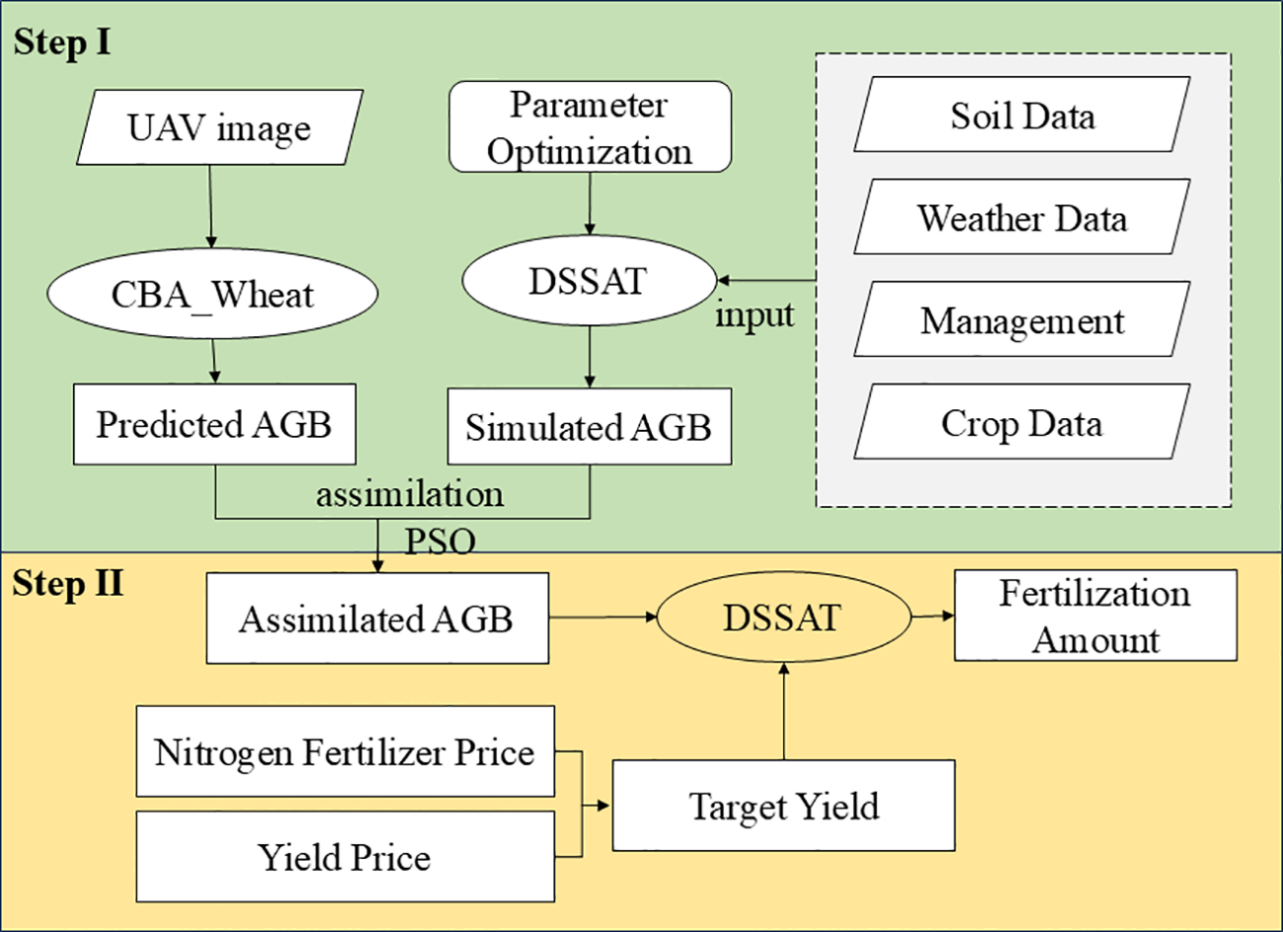
Flowchart of N fertilization recommendation based on a data assimilation system. AGB, aboveground biomass; PSO, particle swarm optimization.

A data assimilation framework was developed to optimize N fertilizer recommendations by integrating a crop growth model with UAV remote sensing data (**Figure 3**). Particle swarm optimization (PSO) is an efficient algorithm inspired by bird flock foraging behavior. Particles adjust their positions based on individual and group experience, with fast convergence and few parameters, making it widely used for crop model parameter optimization (Yi and Ge, 2005; Elbeltagi et al., 2005). This study uses the PSO algorithm for remote sensing data assimilation in the DSSAT (CERES-Wheat) model to optimize crop parameters and recommend N fertilization. The initial soil nutrient contents, including NO_3_^-^-N and NH_4_^+^-N, were obtained from field measurements and used to initialize the DSSAT model. In this study, variations in management practices were appropriately considered during the data assimilation process to simulate crop responses under different management scenarios within a unified parameter framework. Meanwhile, in the simulation of recommended fertilization, top-dressing parameters at the jointing stage were adjusted to analyze top-dressing amounts and target yields under different nitrogen treatments, thereby optimizing the recommended fertilization rates and accurately reflecting crop growth, the specific steps are as follows: First, AGB estimation of winter wheat was conducted based on UAV remote sensing data. Second, AGB data assimilation modeling was performed using the Particle Swarm Optimization (PSO) algorithm. Third, the assimilation algorithm optimized the DSSAT model using the fertilization plans of each experimental plot to ensure that the simulated yields closely matched the target yields for the respective test fields.

#### 2.6.1 Aboveground dry biomass monitoring model based on UAV remote sensing data

Remote sensing monitoring of AGB reflects dry matter accumulation and is closely related to yield; accurate monitoring is a key basis for achieving precise N fertilization. To address the challenge of applying AGB remote sensing models across the entire growth period, this study employs a hierarchical linear model based on accumulated temperature and vegetation indices, which shows significant advantages in monitoring winter wheat AGB at multiple growth stages (Li et al., 2022; Yue et al., 2023; Zhao et al., 2025). In this study, the calculation formula for the AGB monitoring model based on UAV multispectral imagery as Equation 3:

(3) $$\text{Level }1:AGB=\beta_{0}+\beta_{1}\times \text{EVI}2_{i}$$

where AGB, EVI2_i_, *β*_0_*_j_*, and *β*_1_*_j_* are the aboveground dry biomass, enhanced vegetation index 2 obtained from UAV multispectral imagery at different growth stages, the intercept and the slope of the linear model, respectively.

At the second level, these parameters change according to the phenological stages, and the first-level parameters are automatically refined using the phenological data from the second level, as calculated in Equation 4.

(4) $$\text{Level }2:\beta_{j}={\gamma_{m}}_{0}+{\gamma_{m}}_{1}\times \text{GDD}$$

where GDD represents growing degree day, *β_j_*(j = 0, 1) corresponds to *β*_0_ and *β*_1_ from the HLM, respectively, *γ_m_*_0_ is the intercept and *γ_m_*_1_ is the slope of each GDD.

#### 2.6.2 Data assimilation modeling based on particle swarm optimization

In PSO, a swarm of particles explores a D-dimensional parameter space, with each particle representing a potential solution. Particle positions and velocities are updated based on both individual and collective experience to gradually approach the global optimum (Yi and Ge, 2005; Elbeltagi et al., 2005). In this study, AGB during key growth stages was defined as the state variable for data assimilation. Each particle represents a set of DSSAT model parameters, including four genotype traits (P1D, PHINT, RDGS, SLPF) and three management practices (planting density, irrigation, and fertilization). According to preliminary experiments conducted at the same experimental site and the results of previous modeling studies, the initial velocity in each dimension was set to approximately 10% of the dynamic range of the corresponding variable (**Table 1**, Li et al., 2015).

**Table 1:** Initial values and ranges of calibration parameters or initial data for the DSSAT model.

Variable	Initial values	Ranges	Reference
Plant density (m^-3^)	350	300-400	Li et al., 2015
Irrigation amount (mm)	150	90-240	
Fertilization amount (kg N ha^-1^)	200	0-400	
Photoperiod parameter	50	30-70	
Phyllochron interval parameter	100	90-120	
Root depth growth rate	3.0	2.5-3.5	
Photosynthesis factor	1.0	0.8-1.0	

##### 2.6.2.1 DSSAT model initialization

The model is initially configured using field survey data, default parameters and historical data and are used to conduct biomass simulations during key growth stages. The parameters and structure of the crop growth model are typically set based on existing knowledge, experience or default assumptions and are subsequently refined and optimized through calibration and assimilation processes to improve the predictive capabilities of the model.

##### 2.6.2.2 Particle swarm initialization

The basic assumption is that a swarm of 25 particles (Li et al., 2015; Jin et al., 2017) moves with a certain velocity in a d-dimensional search space. Each particle can adjust its trajectory based on the best point found by itself in the current generation (*p_id*) and the best point found by all particles in the swarm (*p_gd*). In the PSO algorithm, the optimization variable is set as AGB during critical growth stages, and a particle swarm is constructed. Each particle represents a set of parameter combinations, with its “position” corresponding to the current values of the parameters. The starting position and velocity of each particle are determined. The parameters subject to adjustment include four crop genotype characteristics (P1D, PHINT, RDGS, and SLPF) (Li et al., 2015) and three plant management parameters (plant density, irrigation volume, and fertilization quantity) (**Table 1**). The initial position and velocity of each particle are calculated as Equations 5, 6:

(5) $$x_{i}\;=\;(x_{i1},x_{i2},\ldots ,x_{id})$$

(6) $$v_{i}\;=\;(v_{i1},v_{i2},\ldots ,v_{id})$$

where xi and vi represent the initial position and velocity of the i-th particle, respectively.

##### 2.6.2.3 Fitness function construction

A cost function was defined to quantify the differences between AGBr and AGBs. The function-generated fitness value indicated whether the optimization process had reached the ideal set of parameters,as shown in Equation 7

(7) $$J\;=\;\sqrt{\sum_{i\;=\;1}^{m}{\left(\frac{AGBs_{i}-AGBr_{i}}{AGBr_{i}}\right)}^{2}/m}$$

where AGBr, AGBs and m represent aboveground biomass predicted from remote sensing data, aboveground biomass simulated by the DSSAT model, and the number of monitored growth stages, respectively.

##### 2.6.2.4 Particle swarm iterative search

Each particle’s fitness value is computed, and the individual best (pbest) is updated if the fitness value surpasses the historical best. Similarly, the global best (gbest) is updated if the particle’s fitness exceeds the first gbest. The velocity and position (model parameters) of the particle are then updated according to a pre-defined Equations 8, 9:

(8) $$v_{id}^{k\;+\;1}\;=\;v_{id}^{k}\;+\;c_{1}\xi (p_{id}^{k}-x_{id}^{k})\;+\;c_{2}\eta (p_{gd}^{k}-x_{id}^{k})$$

(9) $$x_{id}^{k\;+\;1}\;=\;x_{id}^{k}\;+\;v_{id}^{k\;+\;1}$$

where x_id_ and v_id_ denote the position and velocity of the *i*-th particle in the *d-*th dimension of the parameter space, respectively. The parameters (c_1_) and (c_2_) represent the cognitive and social learning factors, both set to 2.0, which is suitable for almost all cases. ξ and η are random values between 0 and 1, which helps the particles explore the search space and obtain the optimal solution. Detailed information on the PSO parameter settings can be found in the study by Eberhart and Shi (2001).

##### 2.6.2.5 Optimal result output

The DSSAT model is rerun using the updated parameters to simulate AGB, and fitness is reassessed. This iterative process continues until convergence criteria are met, such as a defined error threshold or maximum number of iterations. If the iteration target (100 iterations in this study) is not reached, the updated positions are replaced and the next step is executed. The final parameter set producing the best agreement between simulated and observed AGB is identified, providing reliable estimates with enhanced spatiotemporal resolution.

#### 2.6.3 Optimizing N recommendations by integrating target yield and data assimilation

N application at the jointing stage was adjusted according to the fertilization plans of each plot, ensuring that the DSSAT model optimized via data assimilation produced yields close to the target. N input levels were adjusted within a pre-defined range (e.g. 0–360 kg N ha^−^¹) with a constant increment (e.g. 10 kg N ha^−^¹), and the model was run iteratively using the optimized parameters from the previous data assimilation step. For each simulation, the predicted yield was compared against the economically derived target yield. The N rate corresponding to the lowest input that met or slightly exceeded the target yield was selected as the optimal N recommendation. The field-scale economic benefit was calculated as Equation 10:

(10) $$E\;=\;(Y\;\times \;P_{Y})-(N\;\times \;P_{N})$$

where E, Y, PY, N, and PN represent field-scale economic benefit, yield (kg ha^−1^), the market price of winter wheat (CNY kg^−1^), N fertilizer amount (kg ha^−1^), and the cost of N fertilizer (CNY kg^−1^), respectively. The values of yield and N fertilizer were from local government (https://www.ndrc.gov.cn/fgsj/) and defined as 2.4 CNY kg^−1^ and 2.75 CNY kg^−1^, respectively.

### 2.7 Model evaluation

The DSSAT model was calibrated by adjusting AGB to match observed growth and yield measurements. Model evaluation was based on field experiment data, using a stepwise parameter adjustment approach to minimize simulation errors. During the validation phase, independent experimental data were used to assess model performance, primarily through statistical metrics such as R² and nRMSE, to quantify the agreement between simulated results and observed data, ensuring the model’s applicability and reliability under different growing seasons and management conditions. The model’s performance was quantified by the adjusted determination coefficient (R²) and relative root mean square error (nRMSE), with better performance being indicated by a higher R² and a lower nRMSE. The formulas for calculating R^2^, RMSE, and nRMSE are shown in Equations 11, 12, and 13, respectively.

(11) $${\text{R}}^{2}\;=\;1-\frac{\sum_{\text{i }=\;1}^{\text{n}}({\text{Y}}_{\text{i}}-{\text{Y}}_{\text{i}}^{"})/(\text{n}-\text{p}-1)}{\sum_{\text{i }=\;1}^{\text{n}}{\left({\text{Y}}_{\text{i}}-{\text{Y}}_{\text{i}}^{"}\right)}^{2}/(\text{n}-1)}$$

(12) $$\text{RMSE }=\;\sqrt{\frac{1}{\text{n}}\sum_{\text{i }=\;1}^{\text{n}}{\left({\text{Y}}_{\text{i}}-{\text{Y}}_{\text{i}}^{"}\right)}^{2}}$$

(13) $$nRMSE=\frac{RMSE}{average(Yi)}$$

where n, Y′_i_, Y_i_ and p represent the sample size, estimated value, measured value and number of predictors, respectively. In subsequent sections, R² for the calibration and validation sets are denoted as R²_c_ and R²_v_, respectively; nRMSE for these sets are represented as nRMSE_c_ and nRMSE_v_.

## 3 Results and analysis

### 3.1 Winter wheat AGB estimation results derived from hyperspectral data

**Figure 4:**
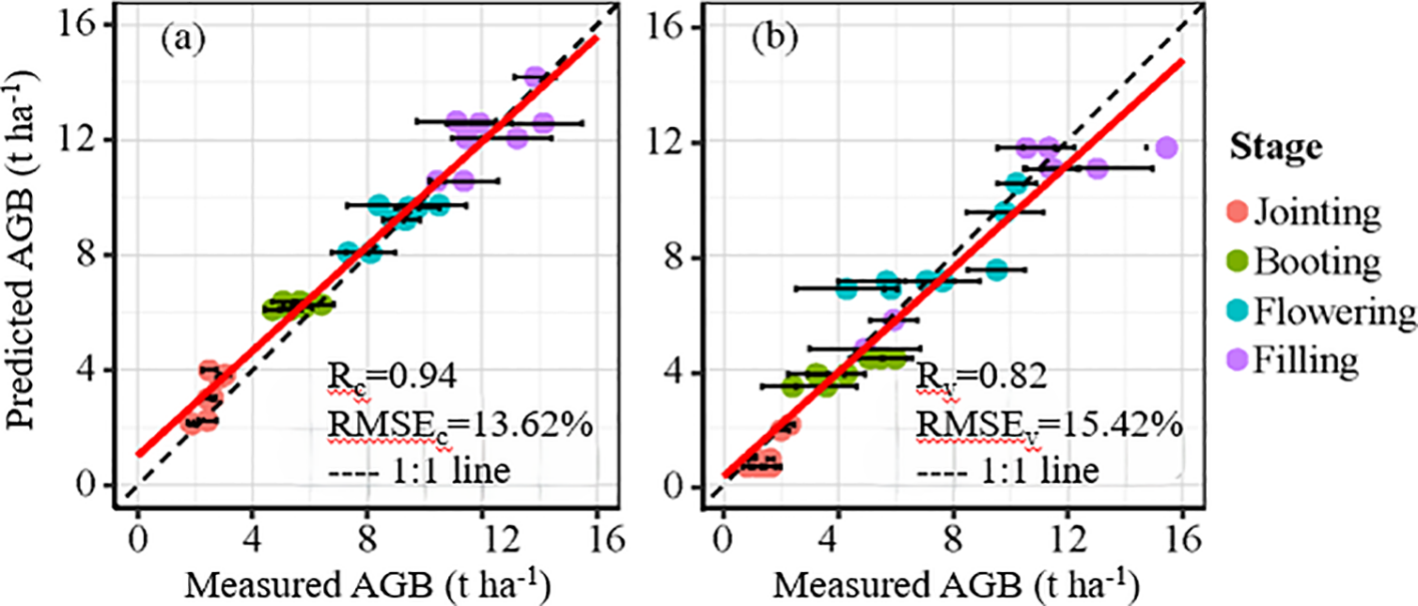
Relationships between measured AGB and predicted AGB by the HLM. The horizontal lines represent the standard error.

In this study, the winter wheat AGB ranges for the training and validation datasets were 1.84–14.14 t ha^−1^ and 0.84–15.44 t ha^−1^, with mean values of 7.27 t ha^−1^ and 5.93 t ha^−1^, respectively (**Figure 4**). To mitigate the effects of growth stages on AGB remote sensing monitoring, this study integrated EVI and growth stage information employing the HLM for winter wheat AGB inversion. Inversion results indicated that the HLM performed excellently, with R²c and R²v values of 0.94 and 0.82 and nRMSEc and nRMSEv values of 13.62% and 15.42%, respectively (**Figure 3**, **Table 2**). These results demonstrate the high accuracy of the HLM in AGB inversion for winter wheat, providing reliable estimates for subsequent N diagnosis.

**Table 2:** Coefficient of each variable in AGB by HLM method.

Parameters	Fixed effect	γ_i0_	γ_i0_	R^2c^	nRMSEc	R^2v^	nRMSEv
AGB	for β_0_	1.22	0.02	0.94	13.62%	0.82	15.46%
	for β_1_	-13.35	0.38				

### 3.2 Evaluation of winter wheat growth process based on DSSAT data assimilation

**Figure 5:**
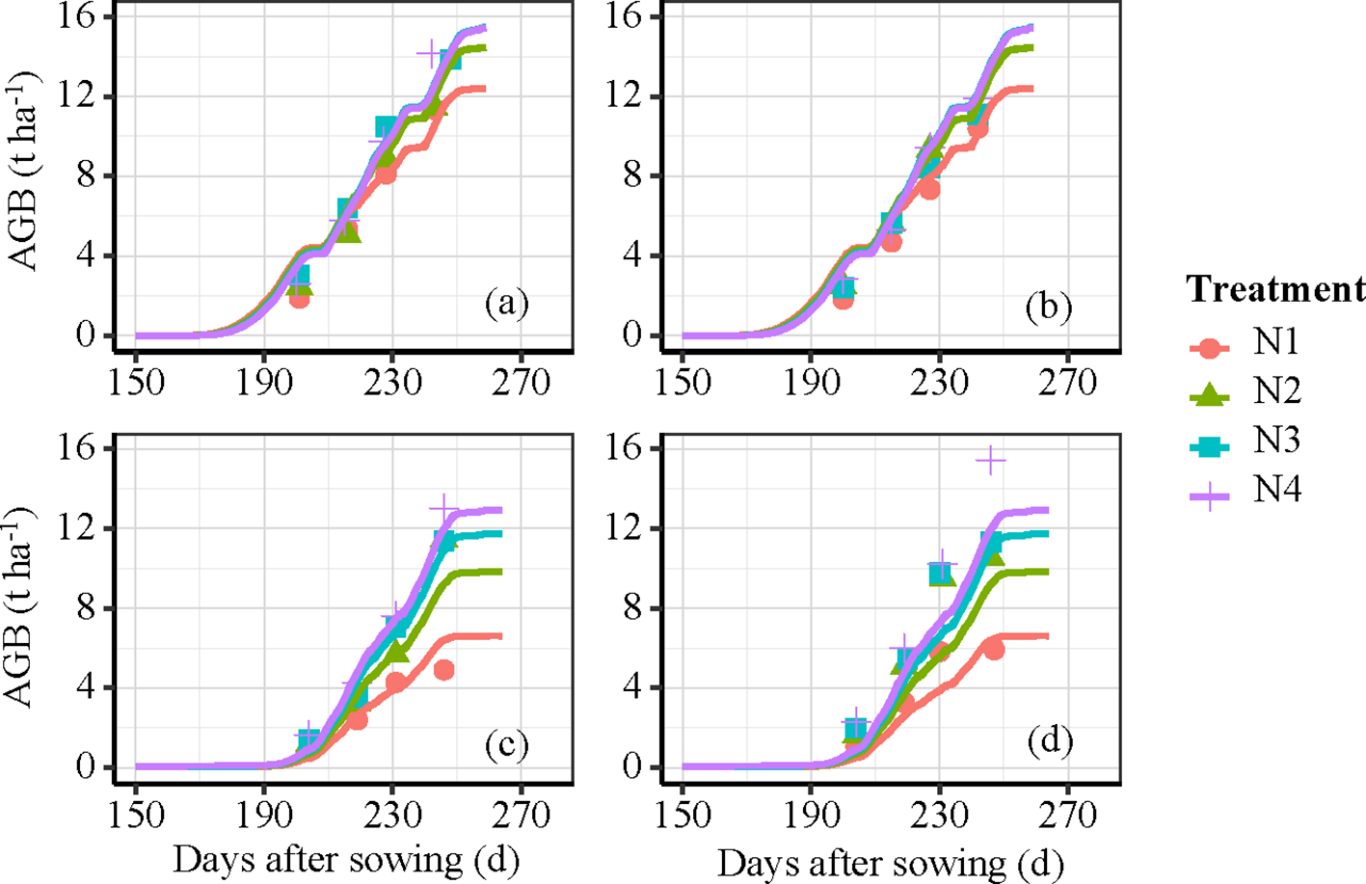
Simulated and measured AGB of winter wheat under different N treatments. Note: Vertical bars are standard deviations of measurements and simulation.

**Figure 6:**
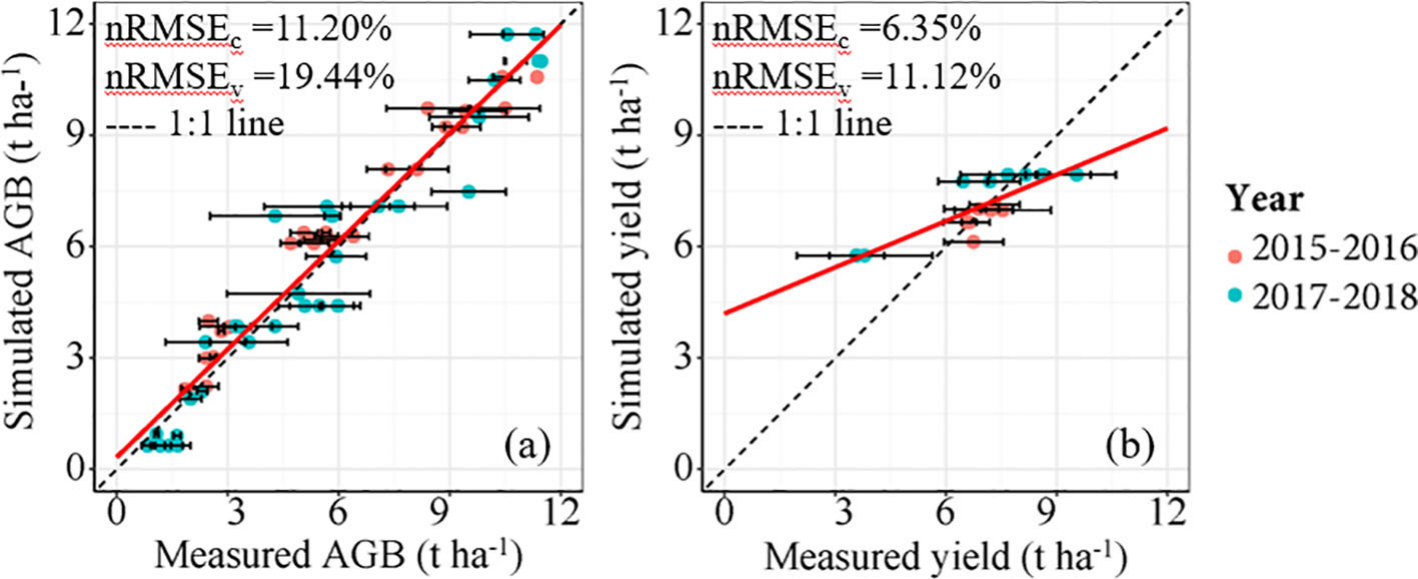
Relationships between measured and simulated AGB **(a)** and measured and simulated yield **(b)** for winter wheat based on DSSAT Model. Note: The horizontal lines in the FIGURE represent the standard error.

The amount of simulated AGB under different N fertilizer treatments based on the DSSAT model increased with the advancement of growth stages. AGB accumulation increased with increasing N fertilizer rates (**Figure 5**), while the difference between N3 and N4 treatments was relatively small. This suggests that excessive fertilization has a limited effect on crop yield improvement. Therefore, N fertilizer recommendations should balance both economic and ecological benefits. The amount of simulated AGB at various growth periods closely corresponded to the measured AGB, with nRMSE values of 11.20% and 19.44% for the calibration and validation datasets, respectively (**Figure 6a**). Similarly, the yield results simulated by the DSSAT model showed good agreement with the observed data, with nRMSE values of 6.35% and 11.12% for the calibration and validation datasets, respectively (**Figure 6b**).

### 3.3 Economic benefit analysis under varying target yield scenarios

**Figure 7:**
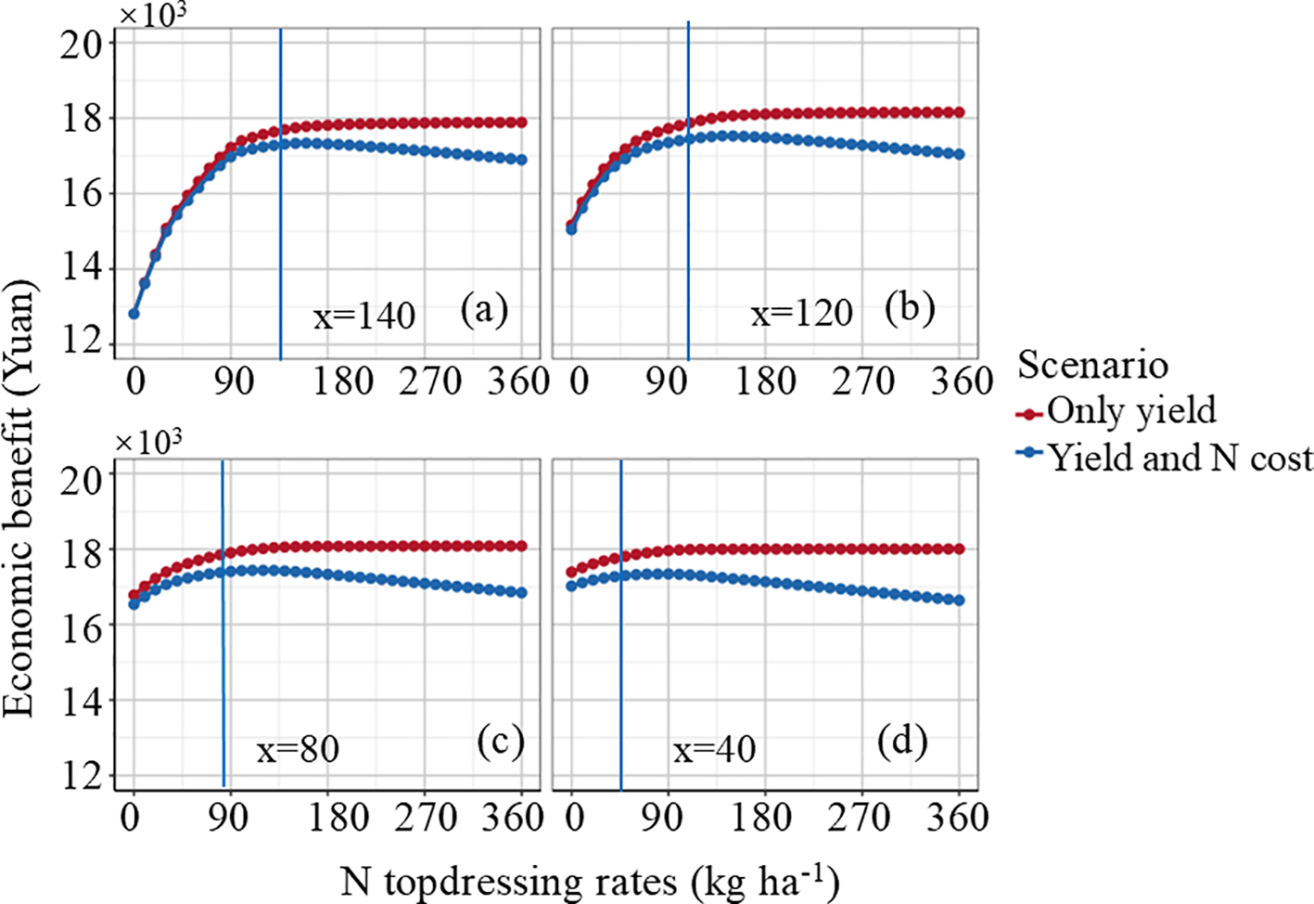
Optimization of N fertilizer for maximizing crop yield and farmer’s net income. **(a)**, **(b)**, **(c)**, and **(d)** represent the base fertilizer treatments as N1, N2, N3, and N4, respectively.

This study explores how crop income responds to different N topdressing rates under fixed basal N levels (N1–N4), with total N topdressing rates ranging from 0 to 360 kg ha^-^¹ (**Figure 7**). When fertilizer costs are not considered, total income increases with the amount of fertilizer applied, indicating that higher N application can boost crop yield and, consequently, total income. Specifically, the total income reached its maximum at a fertilization rate of 360 kg ha^−^¹ under N1, N2, N3 and N4 treatment levels. However, maximizing income without considering fertilizer costs does not reflect the actual profit for farmers. Specifically, the optimal fertilization rates for N1, N2, N3 and N4 treatments are 140 kg ha^−1^, 120 kg ha^−1^, 80 kg ha^−1^ and 40 kg ha^−1^, respectively (**Figure 7**). In practical agricultural production, farmers should find a balance between fertilizer application and its cost to optimize net income. Therefore, the study highlights that while increasing fertilizer application can enhance crop income, excessive fertilization may lead to high costs that reduce net income, making it crucial for farmers to use an appropriate amount of fertilizer to maximize profitability.

### 3.4 N recommendation developed by integrating remote sensing data into the DSSAT model

**Figure 8:**
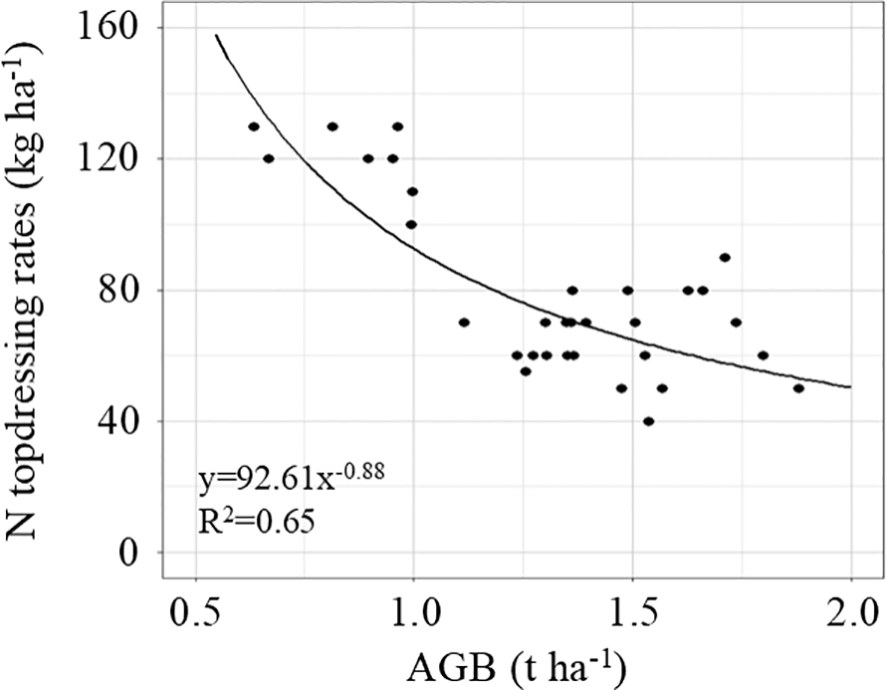
Relationship between AGB and N fertilization topdressing rates based on the data assimilation system.

**Figure 9:**
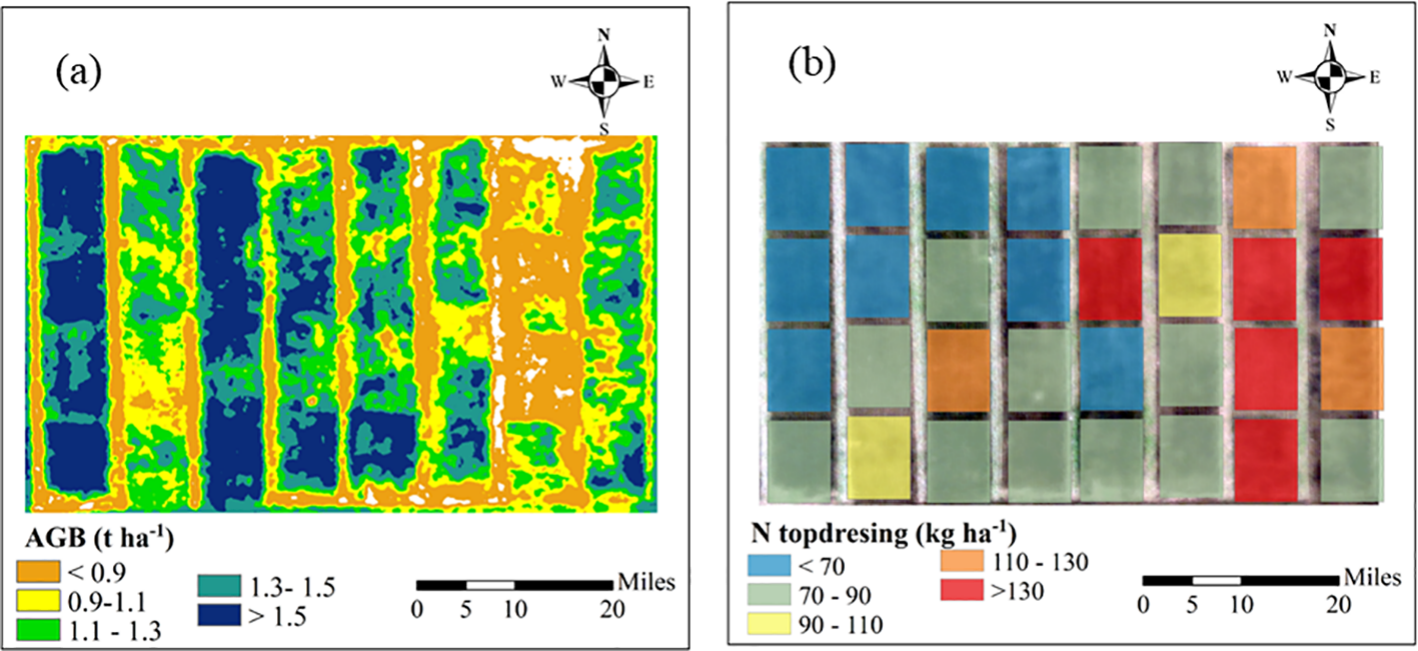
AGB prediction **(a)** and the corresponding fertilization recommendation **(b)** in the topdressing zone using a data assimilation system.

The N topdressing rate recommended by the data assimilation system are primarily determined based on the growth status of the crop and exhibits a significant correlation with AGB at the jointing stage (**Figure 8**). This relationship is described by a quadratic function y = 92.61x^-0.88^, with R² of 0.65. A relatively low AGB indicates weak crop development, necessitating a higher N rate. The spatial distribution maps of AGB and N topdressing rates based on UAV remote sensing data and DSSAT model are shown in **Figure 9**. The results reveal the spatial variability of crop growth status and nutrient requirements in the study area, providing data support for the implementation of variable-rate fertilization management.

### 3.5 Assessment of optimized N topdressing rate performance

**Figure 10:**
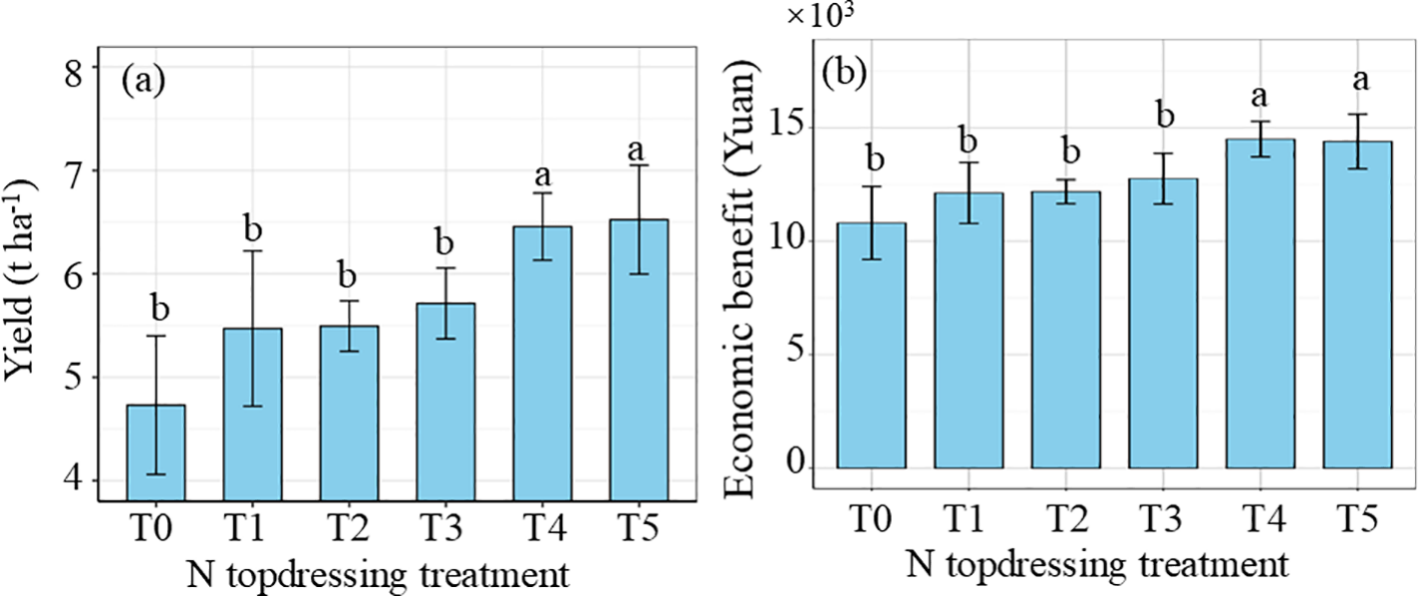
Effects of fertilization treatments on yield **(a)** and economic benefit (Yuan) under different yield levels **(b)**.

In the recommended fertilization trial area (Experiment 3), wheat yields ranged from 3.00 t ha^−^¹ to 8.08 t ha^−^¹. Crop growth simulation models indicate continuous yield generally increases with higher fertilization rates. Therefore, this study selected 125% of the data assimilation-based recommended fertilization amount (T5) as the control treatment. The average yields for treatments T0, T1, T2, T3, T4 and T5 were 4.73 t ha^−^¹, 5.47 t ha^−^¹, 5.50 t ha^−^¹, 5.71 t ha^−^¹, 6.46 t ha^−^¹ and 6.52 t ha^−^¹, respectively (**Figure 10**). The yield of treatment T5 was slightly higher than that of T4 with no significant difference, but the yields of both treatments were significantly higher than those of the other treatments. For economic benefits, treatment T4 slightly outperformed T5 with no significant difference, and both treatments achieved significantly higher economic returns than the other treatments. This suggests that while optimizing fertilization plans, a moderate reduction in N input can still maintain high yield levels, providing a cost-effective and efficient fertilization strategy for agricultural production. In practical applications, appropriate N fertilization not only promotes crop growth but also reduces the risk of environmental pollution.

## 4 Discussions

### 4.1 Significance of AGB remote sensing monitoring in N fertilizer management

Data assimilation algorithms not only offer clear advantages in estimating the current crop state but also demonstrate strong robustness and dynamic adaptability in forecasting later growth stages. This research employs AGB as the assimilation variable, which is vital for formulating accurate fertilization strategies. Li et al. (2024) also employed AGB as a state variable for data assimilation in N recommendation for rice. The AGB monitoring model based on HLM achieved calibration and validation R² values of 0.94 and 0.82 with nRMSE of 13.62% and 15.42% and provided notable scalability throughout the growing season (**Figure 2**, **Table 2**). The jointing stage, as the key period for winter wheat topdressing, is highly sensitive to management practices. This study demonstrates that the N topdressing rate recommended by the data assimilation system exhibits a negative power-law relationship with aboveground biomass (AGB) at the jointing stage of winter wheat (R² = 0.65), a pattern analogous to the critical nitrogen dilution curve (Justes et al., 1994). This relationship arises primarily because (1) plots with higher AGB have sufficient N accumulation and thus require less additional N input, whereas plots with lower AGB are relatively nitrogen-deficient and require greater N compensation., and (2) spatial heterogeneity in soil nutrients and water results in differences in crop growth potential, necessitating location-specific adjustments of recommended fertilization even under uniform management practices. Previous studies using growth indicators such as leaf area index and nitrogen nutrition index for fertilizer recommendation have also obtained similar results (Xue et al., 2008; Li et al., 2024b). This study developed an empirical curve linking biomass to recommended nitrogen application, but it was constructed using data from a specific experimental field. With the accumulation and optimization of more data, this approach has the potential to evolve into a simple fertilization recommendation tool, serving as a practical alternative to complex data assimilation systems.

### 4.2 Constructing target yield for data-driven N management

The setting of target yields should not solely focus on maximizing yield, as this may lead to excessive use of N fertilizer, resulting in environmental issues such as soil acidification and groundwater pollution (Peng et al., 2010). In this study, target yield is constructed based on economic benefits. The calculation of target yield considers not only the final crop yield but also the relationship between fertilizer costs and yield gains. Data assimilation-based fertilization decisions driven by target yield allow for the balancing of yield, costs and profits, thus maximizing economic benefits. The recommendation to enhance N application in fields with poor crop growth and reduce it in fields with robust growth is in agreement with the results from previous research (Zhang et al., 2022). Additionally, data assimilation-based N recommendation methods facilitate the precise achievement of target yields and quantify N fertilizer recommendations for heterogeneous growth zones within fields (**Figures 7**, **9**). In this study, the optimal fertilization rates for N1–N4 treatments (140, 120, 80, and 40 kg ha^-^¹) follow a trend consistent with previous research (Yang et al., 2022; Li et al., 2024a).

Simulation results indicated that, compared with traditional fertilization strategies, this method maintained relatively stable yields while reducing N application by 6.69%–34.08% (**Figure 10**). Therefore, the target yield-based data assimilation strategy holds potential to become a practically applicable approach for fertilization optimization (Wang et al., 2023). Setting target yields depends largely on historical data and predictive models, which can introduce errors, particularly in the face of significant climate variability. To address these uncertainties and to evaluate the environmental benefits of reduced N application, future research integrating field measurements with modeling approaches is essential for guiding sustainable fertilization practices.

### 4.3 Challenges in N recommendation through data assimilation systems

Significant progress has been made in N recommendation methods using data assimilation systems; however, numerous challenges remain concerning their widespread adoption and practical application in agriculture. This research primarily focuses on growth differences in winter wheat caused by nutrient factors and does not consider other influencing factors such as pest and disease stress. The acquisition and quality control of multi-source observational data are primary issues in current N fertilizer recommendation systems (Lu et al., 2022). The effectiveness of data assimilation heavily relies on the accuracy, frequency and spatial coverage of observational data. Issues such as insufficient temporal resolution, limited spatial precision and meteorological interference limit the precision required for N management (Diacono et al., 2013). The uncertainty in remote sensing observations is introduced into the assimilation process, and misleading parameter corrections may occur when observational errors are combined with model biases (Kang and Özdoğan, 2019). This study realized N fertilizer recommendation applications based on UAV data, offering spatial and temporal flexibility, thereby establishing a technically robust and economically feasible pathway for precision agriculture applications. In the future, integrating the spatial advantages of satellite remote sensing data will further enhance the cost-effectiveness and practicality of large-scale precision agriculture applications.

This study not only validates the potential of data assimilation in crop N management but also identifies the directions for the further improvement of model reliability. However, the predictions of a single crop growth model are inherently uncertain (Huang et al., 2017; Zare et al., 2024). The combination of multiple models can effectively address the shortcomings of a single model’s adaptability in specific scenarios, further advancing the application and development of data assimilation technology in agricultural management (Huang et al., 2017; Zare et al., 2024). Existing research indicates that model integration or weighted averaging methods can reduce biases that may arise from a single model. Among studies comparing algorithms that include PSO, most show that PSO outperforms other methods in terms of convergence speed, computational efficiency, and assimilation accuracy. However, some studies report that PSO is not the best-performing algorithm, indicating that algorithm performance depends on the specific study context, assimilation variables, and model complexity (**Table 3**). This study selected the PSO algorithm as the data assimilation method, which has demonstrated good performance in assimilating initial input parameters and simulating grain yield (Wang et al., 2024). The PSO algorithm can operate with a simple encoding scheme and achieves higher retrieval accuracy for nitrogen application rates (Wang et al., 2014). In this study, PSO was applied for fertilizer recommendation, yielding stable results. Future research should compare fertilization strategies developed using multiple assimilation algorithms to identify more accurate assimilation methods for more rational fertilization strategies.

**Table 3:** Comparison of data assimilation algorithms, target variables, and performance in previous studies.

No.	Crop growth model	Crop	Data assimilation algorithm	Variables	Main results	Reference
1	Ricegrow	rice	PSO, SCE-UA	LAI, LNA	PSO showed higher efficiency and better assimilation performance than SCE-UA.	Wang et al., 2014
2	Aquacrop	Winter wheat	PSO, SCE-UA, SA	AGB	PSO, SA, and SCE-UA all simulate winter wheat AGB well, with SCE-UA achieving the highest accuracy and computational efficiency	Xing et al., 2017
3	ChinaAgrosys	Winter wheat	PSO, SCE-UA	Yield	The PSO algorithm achieves the highest accuracy in yield estimation.	Jin et al., 2022

LAI, LNC, AGB, PSO, SA and SCE-UA represent leaf area index, leaf nitrogen concentration, aboveground dry biomass, particle swarm optimization, simulated annealing and shuffled complex evolution.

## 5 Conclusion

This study established an N fertilization decision-making model based on a data assimilation system and conducted detailed evaluation and discussion. The main results are presented below: (1) the AGB monitoring method was developed using GDD and vegetation indices, achieving satisfactory results in both the calibration and validation datasets, with R² (nRMSE) values of 0.94 (13.62%) and 0.82 (15.42%), respectively. (2) The data assimilation stability for AGB and yield is relatively high based on the data assimilation system. For AGB, the nRMSE values are 11.20% and 19.44% for the training and validation datasets, respectively, and for yield, they are 6.35% for the training dataset and 11.22% for the validation dataset. (3) The data assimilation-based recommended fertilization system shows a negative power-law relationship with AGB at the jointing stage (R² = 0.65). Under different yield levels, fertilization was reduced by 6.69%–34.08% compared with that under high yield levels. Data assimilation-based recommended fertilization system proves to be effective, enhancing resource utilization and fostering more sustainable agricultural practices.

## Data Availability

The original contributions presented in the study are included in the article/supplementary material. Further inquiries can be directed to the corresponding author.
